# Should psychology reappropriate the Attention-Seeking?

**DOI:** 10.3389/fpsyg.2026.1732206

**Published:** 2026-02-18

**Authors:** Sebastien Porcher

**Affiliations:** BASF, Ludwigshafen am Rhein, Germany

**Keywords:** Attention-seeking, DSM-5, personality disorder, belongingness, Maslow, socialization, narcissism

Baes et al. (2023) recently highlighted the importance of semantics in naming mental health concepts. Building on this initiative, we reviewed the Personality Disorders in the DSM-5 and identified the term “Attention-Seeking” as ambiguous in its definition (American Psychiatric Association, 2013). The APA describes a pathological psychological trait as “engaging in behavior designed to attract notice and make oneself the focus of others' attention and admiration,” a definition that does not explicitly indicate excess and could apply to “non-pathological behaviors” such as wearing makeup, tattooing, following fashion trends, extravagance, or displaying wealth.

Given this observation, and supported by the arguments presented below, along with the growing discussion regarding the definition of mental illness (Maté and Maté, 2022), we encourage the scientific community to consider classifying Attention-Seeking as a standard psychological trait rather than a psychiatric condition, similarly to the approach taken by the World Health Organization (2022) for the ICD-11, which does not address this concept (Baumeister and Leary, 1995).

## The Attention-Seeking as a standard psychological need

### Integrating into Maslow's hierarchy of (psychological) needs model

By classifying Attention-Seeking (i.e., “Need for Others' Attention”) as a standard psychological trait, and by associating it with a “Social Source” parameterization, this psychological trait offers a backbone for all psychological Needs of the Maslow's model (Maslow, 1943): Love and Belongingness, Self-Esteem, and Self-Actualization.

Under this framework, the Need for Love and Belongingness (the first level of Maslow's pyramid) would be regarded as primarily fulfilled through attention from parents and family, and as this need grows, extended to increasingly distant groups, from friends to the anonymous crowd. Self-Esteem (i.e., Attention from the Self), the next level of the pyramid, reflected in Distinctiveness (Leonardelli et al., 2010) would be sought when individuals perceive that the attention they receive from their group falls short of what they believe they deserve. At the highest level, Self-Actualization (D'Souzy and Gurin, 2016) involves individuals seeking transcendental attention from a higher entity, driven by a unique sense of purpose or spiritual beliefs (Watson et al., 1995; Sevinç and Karatas, 2021).

This thus embedded “social distancing from the source of fulfillment”—ranging from parents to humanity—links all psychological needs in Maslow's Hierarchy of Needs model and creates a continuum within the model, enabling “emotional movement” from one source of fulfillment to another. This feature was recognized by Maslow himself as missing from his original model (Fallatah and Syed, 2017).

### Shedding new light on the socialization process

The “Social Source Distancing” parameterization of Attention-Seeking can also be viewed as a framework for psychological development in humans. Toddlers instinctively seek the attention of their caregivers, which forms the basis for attachment development (Peluso et al., 2004). As children, driven by an increasing need for attention, they turn to groups that are progressively more distant—from family to friends—participating in these interactions as part of their socialization (Bowlby, 1988). Adolescents continue this quest for others' attention, unless discouraged by overvaluation from parents (Piff, 2014; Brummelman et al., 2015; Pleux, 2002). On their path to adulthood, they eventually turn inward to seek attention from the Self, which is essential for building distinctiveness and attracting partners (Tornhill and Gangestad, 1999).

### Giving ground for personality disorders

The “normalization” of Attention-Seeking does not negate its pathological expressions; rather, it rationalizes them as stemming from a common excessive need for attention.

Using the above parametrization, the Histrionic Personality Disorder would be associated with an excessive Need for Attention from the “loves ones” (inner circle), borderline and antisocial behaviors with attention from the crowd [in line with Guilé et al.'s (2016) and Wang et al.'s (2023) views] and the narcissism,^1^ with Attention from the Self, further justifying their B-Clustering by the APA [Cluster B “emotional or erratic behaviors” (American Psychiatric Association, 2013).

### Supporting the concept of “healthy” narcissism

By establishing a continuum of the Need for Attention that spans from healthy to pathological expressions, the still marginalized theory of “healthy” narcissism (Sedikides et al., 2004; Solan, 2016) can gain new validation and serve as a basis for explaining the observed rise in narcissism in Western countries (Twenge, 2013). This epidemic (Twenge et al., 2014) would now be understood as a shift in Attention-Seeking behavior, transitioning from a “healthy” form to a pathological one, driven by a societal culture that prioritizes individualism over collectivism (Humphrey and Bliuc, 2022) and giving an easy access to technologies such as smartphones that facilitate narcissistic behavior (Furinto et al., 2023; Casale and Branchi, 2020).

## Footnotes

1 In its last revision of DSM-5 in 2013, the APA Committee envisaged to remove the Narcissism from the list of personality disorders (Sokodol et al., 2011).
